# The Prevalence and Impact of Allergic Rhinitis on Academic Performance and Quality of Life Among Medical Students in Saudi Arabia

**DOI:** 10.7759/cureus.42342

**Published:** 2023-07-23

**Authors:** Zohour A Almalki, Ayman A Atalla, Fai M Altalhi, Farah S Alnemari, Wurood G Alharbi, Jumana A Almajed, Ayman R Baiuomy

**Affiliations:** 1 Medicine, Taif University, Taif, SAU; 2 Family Medicine, Taif University, Taif, SAU; 3 Medicine, King Abdulaziz University, Jeddah, SAU; 4 Pharmacology, Taif University, Taif, SAU

**Keywords:** education., academic performance, students, rhinitis, allergic

## Abstract

Background: Allergic Rhinitis (AR) has a negative impact on both patients and society. Our study aims to assess the impact of allergic rhinitis on the academic performance and quality of life of medical students in Saudi Arabia.

Methodology: A cross-sectional survey targeted medical students in Saudi Arabia; a total of 851 students were included. The survey included questions on sociodemographic characteristics, academic year and GPA, Score for Allergic Rhinitis (SFAR), and Rhino-sinusitis Disability Index (RSDI).

Results: We found that about 340 students (39.9%) had AR. The RSDI for students with AR was 34.9 ‡ 28.8, significantly higher than those with no AR (17.0 + 23.6),p<0.001. The relationship between AR and students* GPAs showed that those who had not experienced AR significantly had comparatively good PAs and above. In contrast, those who had experienced AR had poor GPAs (p<0.001). Similarly, students with AR had significantly missed classes more than those who had not experienced AR (p<0.001).

Conclusion: The study showed that Allergic rhinitis negatively impacted Medical Students' academic performance and quality of life. It impacts wakefulness, and sleeping patterns can have a negative impact on academic performance.

## Introduction

Allergic rhinitis (AR) is an inflammatory disorder induced by an immunoglobulin E-mediated reaction following allergen exposure. Studies indicate that the prevalence of AR is rising in developing countries. It is estimated that 600 million people globally have AR. Moreover, 200 million of them have bronchial asthma [1]. AR has a significant negative impact on both patients and society [1]. It may interfere with nighttime sleep and cause daytime sleepiness, university absenteeism, mood swings, and other psychosocial issues [2]. All of this impacts academic performance. According to (ISAAC) studies, the frequency of allergic rhinitis in adolescents and children varies significantly worldwide. It may affect 15% of all children between the ages of 6-7 and up to one-third of the population between the ages of 13 and 14 [2]. The typical symptoms of the disorder are rhinorrhea, nasal obstruction, sneezing, and nasal itchiness. A thorough history, physical examination, and allergen skin testing are all necessary to diagnose allergic rhinitis [3]. The AR Impact on Asthma (ARIA) guidelines categorize AR based on the severity and duration of the condition. Depending on the symptoms ' seriousness, they can be categorized as mild, moderate, or severe. According to the duration of the symptoms, it is divided into intermittent or persistent groups [1]. The mainstay of treatment is second-generation oral antihistamines and intranasal corticosteroids [3].

In 2022 a cross-sectional study was conducted to estimate the prevalence of allergic rhinitis and its effects on the QoL among students at a Syrian Private University. It established that 115 (31.1%) students had been diagnosed with AR. Total absences from school were 12.1%, while reduced academic output was 21.8% [4].

Another cross-sectional study has been performed among Free State University (UFS) medical students to determine the prevalence of allergic rhinitis using a self-administered anonymous questionnaire. Data was gathered on the effects of the condition on participants in terms of symptoms, quality of life, disease management, and treatment. A prevalence of 39.1% was found, and sneezing, rhinorrhoea, and nasal obstruction were the most common and bothersome symptoms in those students [5].

Furthermore, a study has been conducted among medical students of Thammasat University to estimate the prevalence and how it affects the QoL using the rhinoconjunctivitis QoL questionnaire (Rcq-36). They found that students who self-reported having rhinitis symptoms within the past year experienced a significant worsening in their quality of life, as measured by the Rcq-36. Eye problems were noticeably more prevalent in those with AR compared to the non-AR group [6].

To our knowledge, no study has been done in Saudi Arabia to assess the impact of allergic rhinitis on the academic performance and quality of life of medical students in Saudi Arabia by using a validated score for allergic rhinitis questionnaire (sfar) and rhinosinusitis disability index (RSDI).

## Materials and methods

Study design

A descriptive, cross-sectional survey was conducted from December to March 2022 to assess the impact of allergic rhinitis on academic performance among medical students in Saudi Arabia. Participation was voluntary and informed consent was obtained from all participants. The institutional research ethics board approval was obtained from the research ethics committee at Taif University (application number: 44-116) before performing any study procedure. Participants' identities were kept confidential. 

Study participants

The study's population involved all male and female medical students in Saudi Arabia who agreed to participate. At the same time, subjects who study outside of Saudi Arabia, Non-medical students, and who disagree with participation were excluded. 

Sample size

The ideal sample size was estimated based on the population size (20,840) [7]. We used a Qualtrics calculator with a confidence level of 99% and a margin error of 5%, the sample size was calculated to be 646 participants, and we added 15% to account for incomplete responses. Therefore, the total sample was around 745. The questionnaire was sent to 1000 randomly chosen people and received 851 responses.

Data collection and instruments

A predesigned online self-administered electronic questionnaire in English was used to collect the data. The questionnaire contained three main parts: sociodemographic data (age - gender - nationality - area of residence), academic year, and grade point average(GPA). In the second part, we used Score for Allergic Rhinitis (SFAR) questionnaire to diagnose and assess the severity of symptoms [8]. In the last part, we used GPA and Rhino-sinusitis Disability Index (RSDI) to assess the student's academic performance and quality of life; the higher RSDI score indicates poor academic performance and quality of life [9]. The questionnaire was sent to the target sample by 13 data collectors through social media apps (WhatsApp and Twitter).

Statistical analysis

After data were extracted, they were revised, coded, entered into statistical software, and analyzed using SPSS Version 23 (SPSS Inc., Chicago, IL, USA); continuous variables were expressed as mean and standard deviation and analyzed using T-test and one-way analysis of variance. Categorical variables were expressed as percentages and analyzed by Pearson's Chi-square test. Statistical significance is considered if the p-value <0.05.

## Results

Our analysis included responses from 851 medical students from different provinces of Saudi Arabia, where 479 (56.3%) students were of the age group of 21-23 years, 613 (72%) were females, 831 (97.6%) were Saudi citizens, 241 (28.3%) were from the fifth year of study, 247 (40.1%) had GPA of 3.75-4.49 out of 5, and 100 (31.9%) had GPA of 3.00 -3.49 out 4 (Table 1).

**Table 1:** Sociodemographic characteristics GPA = Grade Point Average

		N	%
Age (years)	18-20	159	18.7
	21-23	479	56.3
	24-26	185	21.7
	27-29	19	2.2
	>=30	9	1.1
Gender	Female	613	72.0
	Male	238	28.0
Nationality	Saudi	831	97.6
	Non-Saudi	20	2.4
Region	Al-Bahah	64	7.5
	Al-Jawf	67	7.9
	Al-Qassim	68	8.0
	Asir	46	5.4
	Eastern	27	3.2
	Hail	57	6.7
	Jazan	57	6.7
	Madinah	57	6.7
	Makkah	100	11.8
	Najran	67	7.9
	Northern Borders	88	10.3
	Riyadh	110	12.9
	Tabuk	43	5.1
Academic year	First	47	5.5
	Second	100	11.8
	Third	96	11.3
	Four	147	17.3
	Fifth	241	28.3
	Sixth	165	19.4
	Internship	55	6.5
GPA out of 5 (n=616)	< 2.75	67	10.9
	2.75 - 3.74	101	16.4
	3.75 - 4.49	247	40.1
	4.50- 4.74	80	13.0
	≥ 4.75	121	19.6
GPA out of 4 (n=313)	1.75 - 2.74	16	5.1
	2.75 - 2.99	41	13.1
	3.00 - 3.49	100	31.9
	3.50 - 3.74	77	24.6
	≥ 3.75	79	25.2

When we assessed the SFAR characteristics of participants, it was found that 642 (75.4%) had nose problems, and among them, 336 (47.7%) had accompanied itchy-watery eyes. About 70 (10.9%) had nose symptoms in pollen season, and 112(17.4%) had it perennially. The triggers of the nasal symptoms showed that house dust mites were the most common triggers (63.6%), followed by pollens (29.9%) and Epithelia (25.2%). Among those who had nasal symptoms, about 316 (49.2%) reported a history of allergic status, and 56 (8.7%) tested positive for allergy. About 209 (32.6%) had a previous medical diagnosis of allergy, and 382 (59.5%) had a familial history of allergy (Table 2). For each student, the SFAR based on the number of points for eight items was calculated, and those who had a score of >=7 were considered to have Allergic Rhinitis (AR). It was found that about 340 (39.9%) had AR.

**Table 2:** SFAR characteristics of participants. SFAR: Score for Allergic Rhinitis

		N	%
Nose problems experienced	Sneezing	452	53.1
	Runny nose	523	61.5
	Blocked nose	392	46.1
	None	209	24.6
The nose problem has been accompanied by itchy-watery eyes (n=642)	No	306	47.7
	Yes	336	52.3
Months of the year when nose problem was experienced	January	59	9.2
	February	32	5.0
	March	20	3.1
	April	30	4.7
	May	23	3.6
	June	36	5.6
	July	29	4.5
	August (pollen season)	37	5.8
	September (pollen season)	33	5.1
	October	41	6.4
	November	69	10.7
	December	98	15.3
	Most of the year (perennial)	112	17.4
	I don't remember	23	3.6
Triggers of nasal symptoms	Epithelia (cat, dog)	162	25.2
	Pollens	192	29.9
	Cold weather	33	5.1
	House dust mites	406	63.6
	Weather change	5	0.8
	Other	58	9.0
Perceived allergic status	No	292	45.5
	Yes	316	49.2
	I don't know	34	5.3
Previous positive tests of allergy;	No	543	84.6
	Yes and it was negative	43	6.7
	Yes and it was Positive	56	8.7
A previous medical diagnosis of allergy	No	433	67.4
	Yes	209	32.6
Familial history of allergy.	No	260	40.5
	Yes	382	59.5
History of allergy in father (n=382)	Allergic Rhinitis	57	14.9
	Asthma	72	18.8
	Eczema	49	12.8
	None	210	55.0
History of allergy in mother (n=382)	Allergic Rhinitis	79	20.7
	Asthma	72	18.8
	Eczema	65	17.0
	None	229	59.9
History of allergy in siblings(n=382)	Allergic Rhinitis	98	25.7
	Asthma	101	26.4
	Eczema	91	23.8
	None	210	55.0
Nasal symptoms in the past year, including sneezing, runny nose, and blocked nose when the subject did not have a cold or ‘flu’, in the past year	No I haven’t	488	57.3
	Yes I have	363	42.7
Classes missed due to nasal symptoms (n=363)	0 days	208	57.3
	1-4 days	104	28.7
	5-8 days	37	10.2
	9-14 days	11	3.0
	>15 days	3	.8

The RSDI was calculated for each student, and the mean total score for students with AR was 34.9 ± 28.8, significantly higher than those with no AR (17.0 ± 23.6),p<0.001. It was also found that all three RSDI subdomain scores were significantly higher among students who had AR compared to those non-AR students (p<0.001). When we compared the RSDI scores in AR students between different age groups, no statistically significant differences were observed for total RSDI and subdomains (p>0.05). The RSDI scores were significantly higher among males for three subdomains and total RSDI scores than females (p<0.05). No statistically significant differences were observed for total RSDI and subdomain scores between Saudi and non-Saudi citizens and between different academic years (p>0.05). The RSDI and subdomain scores were significantly lower among students who had a GPA of good and above compared to others (p<0.05) (Table 3). 

**Table 3:** Comparison of RSDI scores between various sociodemographic characteristics. GPA: Grade Point Average

		Subdomains of RSDI							RSDI	
		Physical			Functional		Emotional			
		Mean		SD	Mean	SD	Mean	SD	Mean	SD
Allergic rhinitis (n=851)	No	7.3		8.9	5.0	7.6	4.9	8.1	17.0	23.6
	Yes	15.3		10.5	9.8	9.3	9.8	10.7	34.9	28.8
	P value	<0.001			<0.001		<0.001		<0.001	
Age (n=340)	18-20	15.4	11.1		10.0	9.3	11.0	10.6	36.3	29.8
	21-23	15.7	10.1		9.9	9.3	9.3	10.5	34.9	28.4
	24-26	13.4	10.6		8.6	8.6	8.9	10.4	31.2	27.7
	27-29	21.7	10.7		16.2	14.4	18.7	16.0	56.5	40.0
	>=30	23.2	9.4		16.8	9.1	17.6	12.1	57.6	29.3
	P value	0.092			0.132		0.077		0.092	
Gender (n=340)	Female	14.3	9.3		9.1	8.2	8.9	9.3	32.3	24.9
	Male	18.4	13.1		12.1	11.9	12.4	14.0	43.2	37.9
	P value	0.003			0.013		0.011		0.004	
Nationality (n=340)	Saudi	15.3	10.5		9.9	9.3	9.9	10.7	35.2	29.1
	Non-saudi	14.9	7.9		7.3	6.2	5.7	7.2	27.9	17.5
	P value	0.908			0.383		0.222		0.435	
Academic year (n=340)	First	14.7	10.8		9.1	8.7	9.5	9.7	33.3	27.7
	Second	14.3	9.2		9.9	7.5	10.1	8.5	34.2	23.9
	Third	15.8	9.1		9.9	8.0	9.5	9.1	35.2	24.7
	Fourth	16.2	12.0		10.7	10.6	10.0	11.6	36.9	32.7
	Fifth	15.1	10.5		9.9	9.5	9.4	11.0	34.5	29.5
	Sixth	15.0	11.1		8.9	9.6	9.7	11.3	33.9	30.6
	Internship	14.7	8.7		10.0	9.0	10.8	11.5	35.6	26.7
	P value	0.986			0.975		0.999		0.998	
GPA	Poor	17.5	12.1		12.1	10.7	12.4	12.8	42.3	34.4
	Below average	13.8	9.4		8.6	8.3	8.6	9.4	31.1	25.3
	Average	15.3	9.5		9.0	8.3	8.3	9.0	32.7	24.9
	Good and above	12.3	8.5		7.2	7.6	6.5	7.0	26.1	21.5
	P value	0.034			0.034		0.010		0.009	

**Figure 1:**
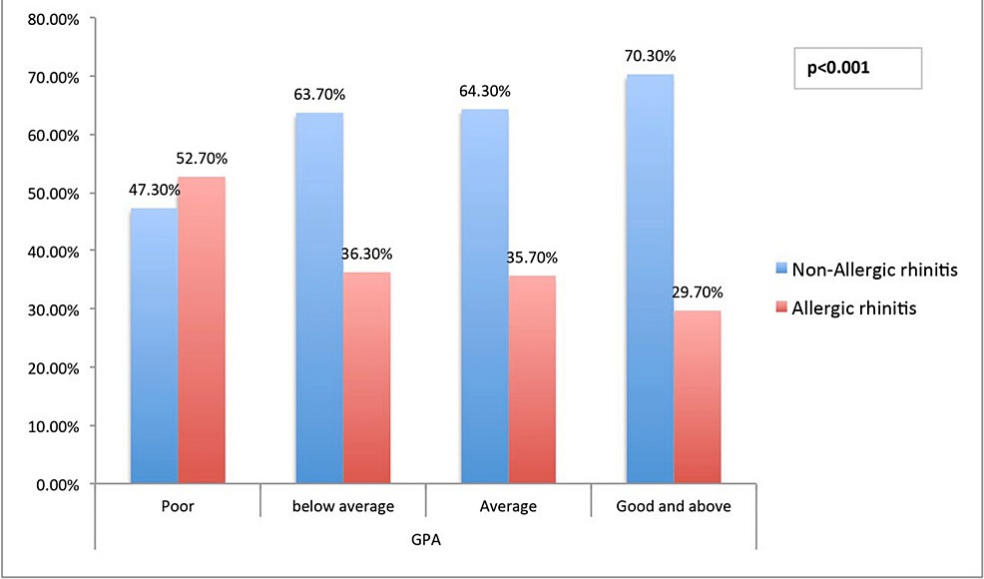
⁨Relationship between Allergic rhinitis and GPA (p<0.001) GPA = Grade Point Average

**Figure 2:**
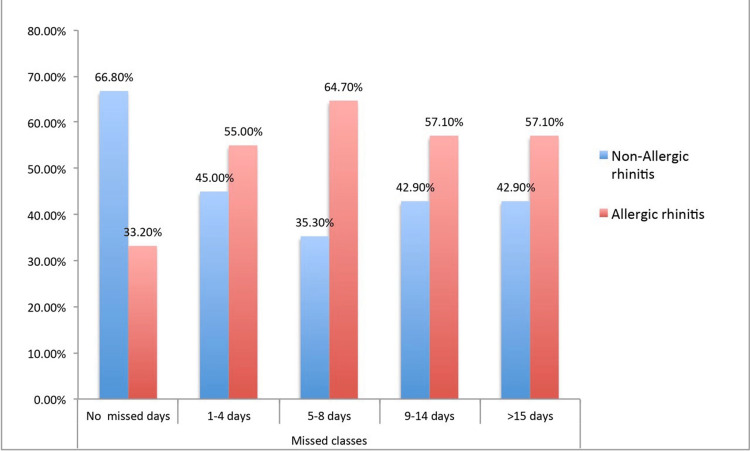
Relationship between Allergic rhinitis and missed academic days (p<0.001)

The relationship between AR and students' GPAs showed that those who had not experienced AR significantly had comparatively good GPAs and above. In contrast, those who had experienced AR had poor GPAs (p<0.001) (Figure 1). Similarly, students who had AR had significantly missed classes more classes than those who had not experienced AR (p<0.001) (Figure 2). A Factorial ANOVA was performed in students with AR by keeping the RSDI score as the dependent variable and other students' characteristics as covariates (Table 4). It was found that the male gender had a significant influence on the RSDI score with a small effect size (F(1,321) =7.48, ηp2 =2.3%, p = 0.007). Also, students' GPA significantly influenced the RSDI with a small effect size (F(1,321) =6.58, ηp2 =2.0%, p = 0.011).

**Table 4:** Factorial ANOVA model for RSDI. GPA: Grade Point Average; RSDI: Rhino-sinusitis Disability Index a. R Squared = 0.049 (Adjusted R Squared = 0.034)

Source	Type III Sum of Squares	df	Mean Square	F	P value	Partial Eta Squared (η_p_^2^)
Corrected Model	13097.464^a^	5	2619.493	3.261	0.007	0.049
Intercept	5415.217	1	5415.217	6.741	0.010	0.021
Age	82.929	1	82.929	.103	0.748	0.000
Gender	6008.358	1	6008.358	7.479	0.007	0.023
Nationality	84.224	1	84.224	.105	0.746	0.000
Current year	692.697	1	692.697	.862	0.354	0.003
GPA	5285.959	1	5285.959	6.580	0.011	0.020
Error	253866.747	316	803.376			
Total	659736.000	322				
Corrected Total	266964.211	321				

## Discussion

Understanding the impact of AR on medical students' quality of life is crucial because of their distinct developmental profile compared to that of younger children and adults and because of the potential impact this has on their academic performance. The findings of this current study showed that the prevalence of AR was 39.9% among the sample of 851 medical students from different regions of Saudi Arabia. Surprisingly, studies from other nations also reported very similar findings [5,8-10]. However, there needs to be more studies in Saudi Arabia, where only one regional study was conducted in Jeddah and reported a prevalence of 16.7% among medical students [11]. The prevalence of AR among the general population in Saudi Arabia ranged from 23 to 48% [1,12-14]. This disparity in AR prevalence could be attributable to variations between regions, groups, or study periods. Indoor and external pollution and diminishing ecosystems might be responsible for the current high prevalence of allergic reactions [15].

Most students believed that their rhinitis was of an allergic origin, but only a tiny percentage had their beliefs confirmed by allergy tests. Dust mites and pollen were the main self-reported causes of most allergic reactions. While many people experienced some degree of symptomatology throughout the year, a considerable proportion of students reported that their symptoms peaked between November and January. This is consistent with previous research showing that winter and spring are when patients complain of AR symptoms [16,17]. About 42.7% of them had reported missing classes, whereas 3.8% had missed classes >=9 days during the academic year, which was statistically significant. Poor academic achievement is more strongly associated with nasal symptoms than eye or asthma symptoms. The current study findings showed that medical students with AR had significantly poor GPAs. Academic challenges were reported by students who suffered from AR and other allergy complaints by Vieira et al. [18]. The authors observed that AR severely hindered the academic performance of 13% of the day's students who experienced the symptoms-up to one-third of reported weeks had recorded time lost from schooling due to allergens. The findings indicate that the primary group of symptoms associated with poor academic performance is nasal symptoms of AR. Finally, immunotherapy has been associated with minimizing the effect of AR on academic performance. These authors' findings emphasize the importance of clinicians paying close attention to the possibility that their younger patients may have AR, which can have a negative effect on a child's performance in school. Moreover, the significance of effectively managing allergy rhinitis in the academic population is also emphasized. Inadequate management of AR and sinusitis have been reported to cause compromised attention and sleep, significantly adversely impacting academic and professional achievement [19,20].

Evidence shows that the majority of students with attention-deficit/hyperactivity disorder (ADHD) are also atopic and experience rhinitis symptoms, such as sleep disturbances, which occasionally might explain the cognitive behaviors seen in ADHD, such as fatigue during the day, apathy, frustration, and impulsivity [20]. This has led to efforts to correlate youngsters' AR with ADHD. Like in any age group, AR can have a significant effect on medical students' or adolescents' quality of life outside of the classroom as well, as it is reported that students with AR and its consequences are at greater risk for developing psychological disorders such as guilt and low self-esteem, as well as family issues such as parental worry, overprotection, and resentment. This might make the student more likely to struggle academically [21,22]. There has been tremendous progress in both the understanding of and remedies for the issue of allergies. It is especially imperative to build trust and good rapport with patients, follow closely and monitor their symptoms during peak pollen season and other times throughout the academic year when symptoms could flare up. The degree to which symptoms can be managed depends on quick symptom identification and treatment plan adaptation. It is essential to plan when treating seasonal symptoms, as many treatments take time to achieve maximum effectiveness after being started. To guarantee that inhalers and nasal sprays are being used correctly, it is essential to conduct regular treatment adherence checks. It has been estimated that after further assessment by an allergist, symptoms and drug use can be reduced by between 50 and 100% [23]. This is due to the allergist's ability to find triggers producing symptoms and provide availability of other avenues for treatment, such as allergen immunotherapy (subcutaneous, sublingual) [24].

Limitations

The current findings emphasize the need for successful treatment of rhinitis among students and the significance of maintaining excellent rhinitis control. Since only a subset of the students had their allergy verified with allergy tests and we did not conduct clinical assessments or allergy tests on the students, we can only presume that they suffered from allergic rhinitis. Although allergic rhinitis is the most prevalent cause of chronic rhinitis, it is conceivable that individuals have another reason. More study is needed to learn how the quality of life is affected by allergic rhinitis and how to manage it in the general population.

## Conclusions

The study showed that Allergic rhinitis significantly negatively impacted medical students' academic performance and quality of life. Male students were found to be more affected due to Allergic rhinitis compared to female students. Currently, allergic rhinitis is one of the most prevalent chronic allergic conditions in young adults, and based on our findings, it could be speculated that wakefulness and disturbed sleeping patterns due to this condition may negatively impact academic performance. Implementing comprehensive management strategies to control and minimize the impact of Allergic Rhinitis can significantly enhance academic performance in affected students by reducing the burden of symptoms and improving overall well-being.
